# A Perplexing Plexopathy After Pembrolizumab Therapy in Early-Stage Triple-Negative Breast Cancer

**DOI:** 10.3390/curroncol33020125

**Published:** 2026-02-20

**Authors:** Toluwalogo Baiyewun, Brian McNamara, Emily Aherne, Alex James Bryan, Julie Twomey, Sorcha NiLoingsigh, Bolanle Ofi, Derek Power, Seamus O’Reilly

**Affiliations:** 1 University College Cork Cancer Center, Cork University Hospital, T12 DC4A Cork, Ireland; toluwalogo.baiyewun@hse.ie (T.B.); alex.bryan2@hse.ie (A.J.B.); julie.twomey@hse.ie (J.T.); bolanle.ofi@hse.ie (B.O.); dpower@muh.ie (D.P.); 2 Department of Clinical Neurophysiology, Cork University Hospital, T12 DC4A Cork, Ireland; brian.mcnamara@hse.ie; 3 Department of Radiology, Cork University Hospital, T12 DC4A Cork, Ireland; emily.aherne@hse.ie; 4 Blood Transfusion Service Board, St Finbar’s Hospital, T12 Y319 Cork, Ireland; sorcha.niloingsigh@ibts.ie; 5 Cancer Research@UCC, University College Cork, T12 CY82 Cork, Ireland

**Keywords:** triple-negative breast cancer, immunotherapy, immune-related adverse events, autoimmune neuropathies, EMG, nerve injury, plasmapheresis

## Abstract

**Background:** In triple-negative breast cancer (TNBC), the addition of immunotherapy has significantly improved outcomes. Immune-related adverse events (irAEs) can be accelerated in patients with pre-existing autoimmune (AI) conditions. The treatment-response standardized protocol used in clinical care raises concerns about the need for right-sizing strategies. As the use of immunotherapy expands, recognizing toxicity from recurrence and optimizing response-adapted approaches are essential to balance cure with quality of survival. **Case Presentation:** A 38-year-old pregnant woman with a distant history of uveitis and psoriasis was discovered to have pregnancy-associated TNBC. Postnatally, she was treated with neoadjuvant chemotherapy and pembrolizumab, followed by wire-guided left breast wide local excision and sentinel lymph node biopsy of the left axilla. After surgery, residual cancer was noted. She continued adjuvant pembrolizumab and adjuvant radiotherapy 40.05 Gy/15 fr to the breast and nodes, followed by a 13.35 Gy/5 fr boost to the tumour bed (breast). Despite a persistent residual tumour, pembrolizumab was continued as per protocol in a response-agnostic manner. At the end of one year of adjuvant pembrolizumab, she developed progressive numbness and weakness in the ipsilateral arm, initially raising suspicion for local recurrence. Comprehensive MRI and PET-CT imaging did not identify recurrent tumour or new metastatic disease. Electromyography confirmed a lower-trunk brachial plexopathy without a structural cause. An immune-mediated process was diagnosed by a process of elimination. Despite treatment with 1st-line high-dose corticosteroids and 2nd-line intravenous immunoglobulin (IVIG), improvement was limited. Therapeutic plasmapheresis led to marked functional recovery and symptom resolution 20 months later. **Discussion:** Four main challenges are identified: (1) the diagnostic difficulty in identifying local recurrence or radiation injury from immune-related neuropathy; (2) the emerging therapeutic role of plasmapheresis in steroid-refractory irAEs; (3) the possible inconsistencies between rare toxicities observed in clinical trials vs. clinical practice; and (4) the limitations in response in adjuvant therapy, particularly in patients with coexisting AI conditions. **Conclusions:** Early recognition and accurate distinction from tumour recurrence, as well as support for plasmapheresis as a potential option in steroid-refractory presentations, have been shown to improve patient survival and symptom reduction. With increasing use of immunotherapy, real-world toxicity data, predictive biomarkers, and personalized treatment strategies are urgently needed to balance cure with long-term functional outcomes.

## Simple Summary

Checkpoint inhibitors have been integrated into the guideline management of early-stage triple-negative breast cancer (TNBC). Current guidelines recommend immune checkpoint inhibitors for 1 year, regardless of perioperative treatment response. Biomarkers to aid “right sizing of therapy” rather than “one size fits all” are not available, and this remains an ongoing area of research. For clinicians, diagnostic challenges arise when distinguishing treatment side effects from immunotherapy toxicities. We report a case of ipsilateral immune-mediated brachial plexopathy in a patient with a history of uveitis and active scalp psoriasis. There was initial concern for local recurrence after treatment with neoadjuvant chemoimmunotherapy as symptomatic, diagnostic, and therapeutic challenges arose. Investigational imaging and neurophysiologic assessment excluded structural aetiologies. First-line corticosteroids and second-line intravenous immunoglobulin (IVIG) showed partial improvement. However, following third-line plasmapheresis, significant recovery was seen. This case illustrates the diagnostic challenges seen in neurological immune-related adverse events (n-irAEs). It presents the spectrum of symptomatology and showcases the importance of distinguishing disease recurrence from immune toxicity. It also demonstrates the investigational tortuosity and diverse treatment options, while elevating the beneficial and evolving role of plasmapheresis in refractory irAE presentations. Additionally, it highlights the need for potentially toxicity-sparing biomarkers of response to aid treatment “right sizing” for patients, particularly those with co-morbidities.

## 1. Introduction

Immunotherapy has become a cornerstone in the management of early TNBC [1]. The pivotal KEYNOTE-522 trial demonstrated that combining pembrolizumab with neoadjuvant chemotherapy significantly improves pathological complete response (pCR) (64.8% vs. 51.2%), event-free survival, and overall survival [2]. Adverse events of grade 3 or higher occurred in 12.9% vs. 1.8% of patients, with most occurring primarily during the neoadjuvant phase [2]. Uncertainty remains regarding the benefit of continuing adjuvant pembrolizumab in this patient cohort [3,4]. In real-world practice, such patients complete one year of treatment regardless of tumour response, exposing them to potential overtreatment and immune-related toxicity.

IrAEs are a recognized consequence of immune checkpoint inhibitor (ICI) therapy. In clinical trials such as KEYNOTE-522, which led to the addition of pembrolizumab to neoadjuvant chemotherapy in early TNBC, grade ≥ 3 irAEs occurred in approximately 13% of participants receiving pembrolizumab, with endocrine, gastrointestinal, and pulmonary events among the most common severe toxicities [2,3].

Real-world experience from the Neo-Real/GBECAM0123 cohort further expands this. In that study of 368 early-stage TNBC patients treated with neoadjuvant and adjuvant pembrolizumab plus chemotherapy, 31% of patients experienced any-grade irAEs, and 13.6% experienced grade ≥ 3 events overall [3]. Most irAEs (≈73%) occurred during the neoadjuvant phase, 28.1% during the adjuvant phase, and 16% of all treated patients required permanent discontinuation of pembrolizumab due to toxicity [3]. Endocrine toxicities were the most frequent (12.8%), followed by cutaneous (7.6%) and gastrointestinal (7.1%) events [3].

Most irAEs affect the gastrointestinal, endocrine, skin, or lung systems; n-irAEs are uncommon, with an incidence of about 1–3% in both clinical trials and real-world settings [2,3,4,5]. N-irAEs can affect the central or peripheral nervous systems and mimic other neuromuscular disorders, leading to diagnostic delays and under-recognition. Unlike the niche populations in clinical trials, the broader real-world patient population may show toxicity patterns not seen in pivotal trials. Additionally, patients with pre-existing AI conditions, such as psoriasis, may face a higher risk of developing these irAEs [6,7,8,9,10,11,12].

Among n-irAEs, brachial plexopathy is exceptionally rare [13]. Survivors of breast cancer with symptoms of new-onset ipsilateral neuropathy are initially ascribed to tumour recurrence, radiation-induced fibrosis, or surgical trauma. The management, prognosis, and survival of these differ, necessitating differentiation from immune-mediated inflammation.

Presented is a case of pembrolizumab-associated brachial plexopathy in a patient with TNBC, first suspected to be local recurrence. After thorough imaging and neurophysiological findings, the diagnosis pointed to an immune-mediated process. The patient’s poor response to corticosteroids and IVIG, followed by improvement after therapeutic plasmapheresis, highlights both the diagnostic and therapeutic challenges of n-irAEs with immunotherapy.

## 2. Case Presentation

A 38-year-old woman, gravida 2 para 2, presented during the third trimester of pregnancy (39 weeks) with a palpable mass in the lower outer quadrant at the 6 o’clock position in her left breast measuring 3–4 cm. Her medical background consisted of bilateral idiopathic anterior uveitis and psoriasis (pityriasis amiantacea). Initial ultrasound suggested a benign papillary or lactational lesion. However, a repeat biopsy confirmed grade 3 invasive ductal carcinoma, triple-negative subtype (ER−, PR−, HER2−). Staging CT of the thorax, abdomen, and pelvis, including bone scan, revealed a 6.3 cm primary mass without distant metastases. Following induction of labour and delivery at 39 weeks, she commenced neoadjuvant chemotherapy consisting of weekly paclitaxel and carboplatin, followed by doxorubicin, cyclophosphamide, combined with pembrolizumab in line with the KEYNOTE-522 protocol [2].

Serial clinical assessments after completion of neoadjuvant treatment demonstrated tumour softening and partial radiological response. She underwent wide local excision (WLE) and sentinel lymph node biopsy, which confirmed residual grade 3 invasive ductal carcinoma (5.9 cm, ypT3 ypN0) with tumour extension into the dermis and a residual cancer burden score of 3. Lymphovascular invasion was absent. Adjuvant pembrolizumab every three weeks was continued for one year, and adjuvant radiotherapy of 40 Gy/15 fr/3 weeks to the breast and regional lymph nodes, followed by a boost to the tumour bed (breast) of 13.35 Gy/5 fr/1 week, was delivered to the left breast and associated nodal fields.

**Figure 1:**
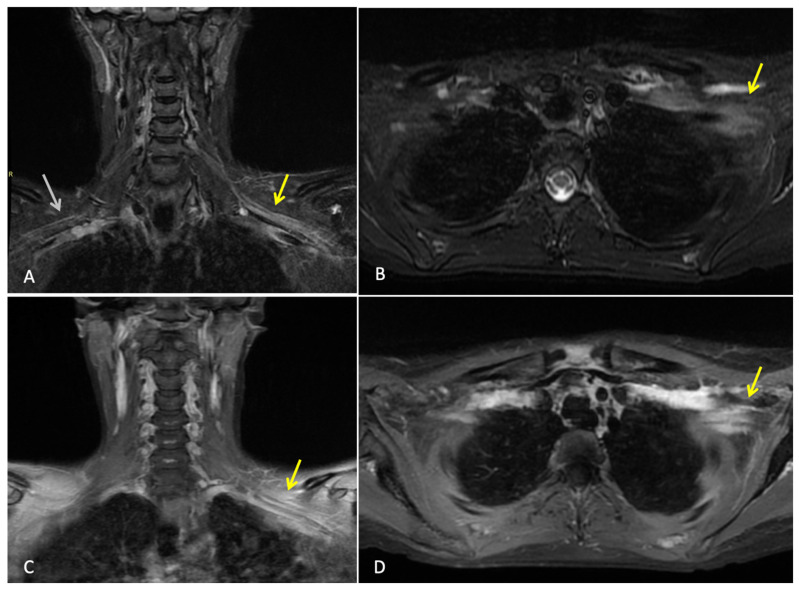
**MRI brachial plexus:** This is a figure. Images (**A**,**B**) are coronal and axial T2 STIR images that demonstrate thickening and increased T2 signal of the brachial plexus on the left (yellow arrows). Normal size and signal of the brachial plexus on the right for comparison (white arrow). Images (**C**,**D**) are coronal and axial T1 fat-saturated post-contrast images, which demonstrate abnormal hyperenhancement of the thickened brachial plexus on the left side (yellow arrows). Findings are consistent with left brachial plexitis.

**Figure 2:**
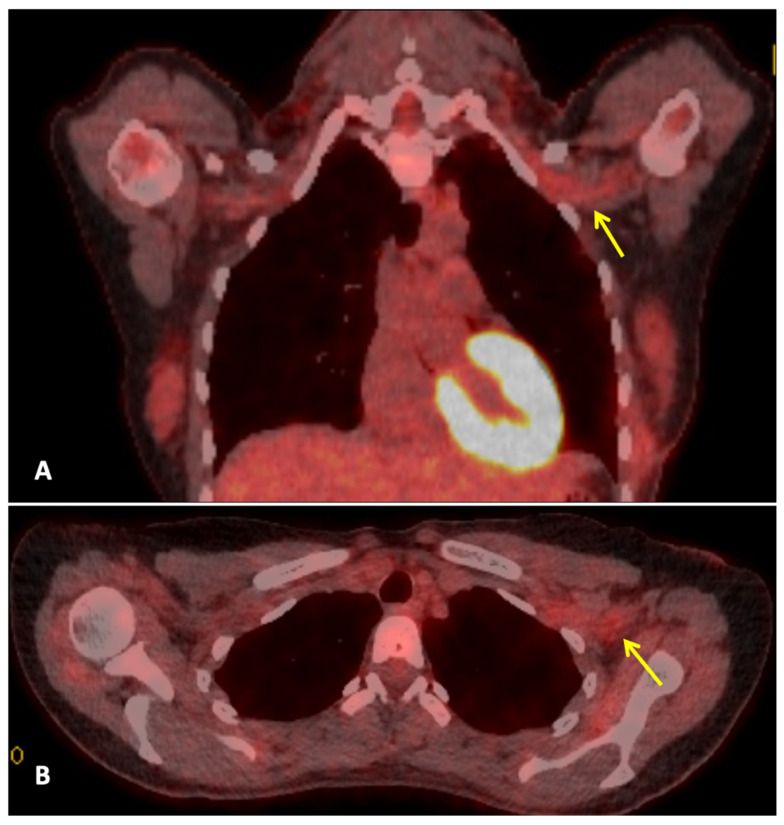
**Whole body FDG PET-CT:** This is a figure. Images (**A**,**B**) are coronal and axial fused PET-CT images of the thorax. They demonstrate asymmetric low-grade uptake in the left brachial plexus (yellow arrows) compared to the right side, but there is no soft tissue mass or high-grade uptake to suggest metastatic disease.

Three months after completing radiotherapy, she developed progressive numbness, paraesthesia, and weakness of the left hand. Examination revealed wasting of the thenar and hypothenar eminences, reduced grip strength, and patchy sensory loss in a C8/T1 distribution. Due to residual cancer burden after surgery, a local recurrence was initially suspected. MRI of the cervical spine and brachial plexus (Figure 1) did not demonstrate metastatic disease, but findings were consistent with brachial plexitis. No recurrent disease was identified on PET-CT, but asymmetric low-grade uptake in the left brachial plexus was also consistent with brachial plexitis (Figure 2), confirming the absence of malignancy. Electromyography (EMG) (Table 1 and Table 2) and nerve conduction studies demonstrated denervation consistent with a lower-trunk brachial plexopathy; onconeuronal antibody serologies were negative.

**Figure 3:**
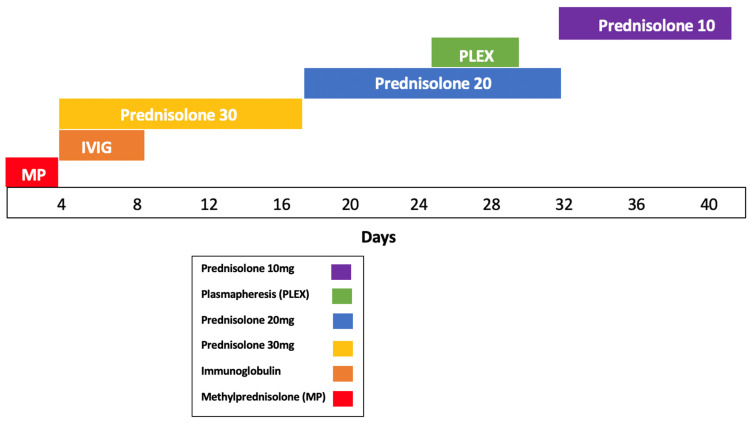
Timeline of immunomodulatory therapy.

Initial EMG showed abnormalities in the radial, ulnar, and median nerves, supporting the diagnosis of immune-related brachial plexopathy secondary to pembrolizumab—her first treatment involved high-dose intravenous methylprednisolone 1 g/day for 3 days, followed by an oral taper of prednisolone with minimal improvement. IVIG therapy (20 g over 4 h for 5 days) yielded partial recovery in grip strength and neuropathic pain, and fine-motor weakness. Although two lines of treatment were administered, symptoms remained functionally limiting. Therapeutic plasmapheresis (5 procedures over 5 days) was performed, resulting in further functional improvement and objective EMG recovery (Figure 3). A follow-up EMG demonstrated normalization and resolution of motor and sensory conduction, confirming electrophysiological resolution of the plexopathy (Table 1 and Table 2).

**Figure 4:**
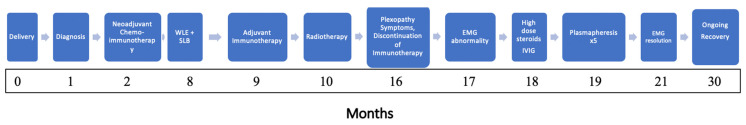
Timeline of oncologic treatment and immune-related neurotoxicity.

After three lines of treatment, she reported marked improvement in grip and dexterity with mild paraesthesia and minimal motor fatigability. Near-complete strength and sensory recovery, consistent with nerve regeneration, were evident on reassessment. She developed worsening psoriasis requiring apremilast, a PD-4 inhibitor. The patient remains in remission 30 months post-surgery (Figure 4).

## 3. Discussion

Four major clinical challenges are presented from this case. Firstly, the newly developed ipsilateral neurological deficits closely mimicked locoregional recurrence, necessitating urgent and extensive investigation. Secondly, challenges from potentially overlapping differential diagnoses, including tumour recurrence, radiation-induced injury, paraneoplastic syndromes, and immune-mediated neuropathy. Thirdly, the patient’s neurological toxicity was unresponsive to corticosteroids, requiring escalation to IVIG and ultimately plasmapheresis. Finally, this case raises important questions regarding the real-world burden of toxicity associated with adjuvant pembrolizumab. This is particularly seen in patients with incomplete pathological response, and those with comorbidities, where therapeutic benefit is not certain and treatment-related morbidity may significantly affect long-term quality of life [2,3,14,15].

### 3.1. Toxicity Burden of Immunotherapy in Early TNBC

Integration of ICIs into neoadjuvant treatment algorithms for TNBC has improved pathological complete response (pCR) rates, as demonstrated in the KEYNOTE-522 trial [2]. However, new data emerging from the real world show a greater prevalence, severity, and persistence of irAEs than controlled clinical trials. In the Neo-Real/GBECAM0123 cohort, Andrade et al. reported that 31% of patients experienced irAEs, with 13.6% developing grade ≥ 3 events; notably, 28% of irAEs occurred during the adjuvant phase, and 16% of patients permanently discontinued pembrolizumab due to toxicity [3]. Similarly, Jayan et al. reported irAEs in 34% of patients treated with the KEYNOTE-522 regimen in routine practice, with a substantial proportion requiring hospitalization or permanent treatment cessation [4]. These findings highlight a widening gap between trial-reported toxicity and real-world tolerability.

Several studies have shown that the development of irAEs, reflecting heightened immune activation, may correlate with improved pathological response [15]. However, this association does not mitigate the risk of irreversible toxicity, nor does it justify treatment continuation in patients experiencing severe or disabling adverse events. Importantly, the benefit of continuing adjuvant pembrolizumab remains uncertain not only in patients who fail to achieve pCR but also in those who do achieve pCR, as no randomized data currently define which patients derive ongoing benefit. This question is being actively investigated in de-escalation trials such as NCT05812807. In real-world practice, particularly among patients who develop early or severe toxicity, the balance between potential benefit and irreversible harm remains unclear [14,16]. These uncertainties highlight the urgent need for personalized, response-adapted strategies and predictive biomarkers to guide immunotherapy continuation in early TNBC [14,15,16,17].

### 3.2. Diagnostic Complexity of Immune-Mediated Brachial Plexopathy

In this patient, the presentation of progressing ipsilateral upper limb neurological symptoms (numbness, paraesthesia, and hand weakness) suggested locoregional recurrence, especially given her incomplete pathological response. These symptoms usually point towards locoregional recurrence, radiation-induced brachial plexopathy (RIBP), or metastatic disease. It further required ample imaging and neurophysiological evaluation [18,19,20]. MRI and PET-CT, however, demonstrated no evidence of tumour recurrence, nodal disease, or infiltrative pathology. MRI is integral in differentiating tumour infiltration, radiation-induced fibrosis, and inflammatory plexopathies. The absence of mass effect or structural abnormality in this case reduced the possibility of a malignant or compressive cause. However, electromyography revealed findings consistent with lower trunk brachial plexopathy [18,21].

Tumour-related brachial plexopathy can occur at variable time points following treatment and may present at any stage, although in triple-negative breast cancer, recurrence risk is often highest within the first year. When present, tumour-related plexopathy typically demonstrates a rapidly progressive course with severe pain and imaging evidence of mass effect or infiltrative disease [19]. In contrast, radiation-induced brachial plexopathy generally develops years after radiotherapy and follows a slowly progressive, often painless course with characteristic fibrotic or thickening changes on imaging [18,22].

In this case, symptom onset occurred seven months postoperatively during adjuvant pembrolizumab, with subacute inflammatory progression, absence of mass lesion on imaging, and electrophysiological features inconsistent with radiation injury, favouring an immune-mediated etiology over tumour recurrence or radiation-induced plexopathy.

Furthermore, electrophysiologic features did not suggest the chronic demyelination typical of radiation-induced neuropathy [20]. While genetic radiosensitivity (e.g., ATM mutations) has been implicated in rare cases of severe RIBP, germline testing was negative in this patient [23]. Radiation-induced brachial plexopathy was considered less likely given the short interval from completion of radiotherapy, the subacute and progressive onset of symptoms. Similar characteristic MRI findings of nerve thickening were seen; however, no fibrosis was seen on imaging, which would be characteristic of radiation-induced plexopathy. Electrophysiological features were inconsistent with chronic radiation injury. Taken together, the clinical timeline, mixed imaging findings, and neurophysiologic results adequately support immune-mediated brachial plexopathy as the most plausible diagnosis, favouring an immune-mediated etiology.

### 3.3. Treatment Escalation and the Role of Plasmapheresis

N-irAE from ICI toxicity shows up in about 1–3% of patients, often mimicking other neuromuscular disorders, contributing to diagnostic delay and under-recognition. Although corticosteroids remain first-line therapy, a subset of patients develop steroid-refractory disease requiring additional immunomodulatory intervention [24,25].

Therapeutic plasma exchange (TPE) is an established immunomodulatory therapy used in a range of antibody-mediated and immune-complex–driven neurological disorders [26,27,28]. In the context of ICI toxicity, TPE is a valuable option in severe or refractory cases [5,26]. Plasmapheresis is a procedure facilitating the removal of circulating autoantibodies, immune complexes, cytokines, and potentially immune checkpoint inhibitor–drug complexes [25,26,27,29].

There is a potential role of early intervention with plasmapheresis in severe n-irAEs. Katsumoto et al. reported improved neurological outcomes when TPE was implemented early in patients with steroid-refractory immune-related toxicity, suggesting a critical therapeutic window [5]. Although largely observational, case reports and small series have demonstrated neurological recovery following plasmapheresis in immune-mediated neuropathies, including brachial plexopathy associated with PD-1 inhibitors [5,26,28,30]. We observed meaningful functional improvement after escalation to plasmapheresis, supporting its consideration as rescue therapy when standard immunosuppression fails [25,26].

Randomized controlled data are lacking, and practical considerations—including vascular access, anticoagulation, and resource availability—must be weighed on an individual basis [26,27]. Alternative plasma purification techniques, such as immunoadsorption, double-filtration plasmapheresis, and cascade adsorption, offer theoretical advantages but require further evaluation for use in the IrAE setting [5,25,28,29]. Despite the favourable response observed in this case, the role of plasmapheresis in immune-related neurological toxicity remains uncertain, with current evidence limited to small case series and individual reports. Its use should therefore be regarded as a consideration in carefully selected refractory cases, rather than a definitive therapeutic approach. Nevertheless, this case contributes to the growing literature supporting the consideration of plasmapheresis as an adjunctive strategy in severe ICI-related neurological toxicity.

### 3.4. Right-Sizing Therapy in Early TNBC: Integrating Toxicity, Autoimmune Risk, and Benefit

KEYNOTE-522 demonstrated improved event-free survival with both neoadjuvant and adjuvant pembrolizumab; the benefit from continued adjuvant therapy is not uniform across all patients, particularly in those with incomplete pathological response or early treatment-limiting toxicity [2,14]. Toxicity-related interruption or discontinuation can dampen therapeutic effectiveness, especially in patients with residual disease who have already demonstrated poor neoadjuvant benefit [4,14,16]. Prolonging immunotherapy exposure in patients unlikely to derive incremental benefit risks irreversible morbidity, raising important survivorship concerns.

This risk may be amplified in patients with pre-existing autoimmune disease, in whom immune checkpoint inhibitors exacerbate latent immune dysregulation through heightened T-cell activation and cytokine signalling [7,10,11]. Cutaneous autoimmune conditions such as psoriasis are particularly prone to flare during immunotherapy, often reflecting broader immune activation that may predispose to multi-organ toxicity [6,7]. In the present case, pembrolizumab exacerbated pre-existing psoriasis, requiring systemic therapy. A condition that preceded the development of immune-mediated brachial plexopathy, suggesting early autoimmune destabilization may have served as a clinical harbinger of severe downstream neurotoxicity.

Patients with autoimmune disease may experience higher rates, greater severity, and increased chronicity of immune-related adverse events, frequently necessitating immunosuppression or permanent treatment discontinuation [8,9,11]. In the adjuvant setting where survival benefits may be modest, the continuation of ICI exposes patients to long-term morbidity without a clear survival advantage. This points to the need for predictive tools, response-adapted strategies, and de-escalation frameworks to provide a more personalized approach. Such a need to integrate pathological response, toxicity burden, autoimmune risk, and patient preference to optimize the risk–benefit ratio of immunotherapy in early TNBC has been highlighted by multiple groups [31,32,33,34].

This case demonstrates a clinical conundrum, in which protocol-driven continuation of adjuvant immunotherapy in a patient with pre-existing autoimmune disease resulted in substantial toxicity with uncertain incremental benefit, further magnifying the need for response- and toxicity-adapted treatment strategies.

### 3.5. Survivorship and Long-Term Supportive Care

As survival in early TNBC improves, long-term treatment-related morbidity has emerged as a key factor in an individual’s quality of life [16]. Neurological irAEs, although rare, may result in persistent functional impairment, neuropathic pain, and reduced independence, requiring multidisciplinary care [13,24]. In younger patients, this can impact their employment, caregiving responsibilities, and mental health.

Survivorship care blends toxicity surveillance, rehabilitation, and supportive care into routine oncology practice, particularly for patients with immune-mediated complications [16]. This case highlights prioritization of quality of survival and disease control. Also, reinforcing the importance of early recognition and aggressive management of rare toxicities.

## 4. Conclusions

In summary, this case demonstrates how immune-mediated plexopathy can mimic recurrence and the therapeutic efficacy of plasmapheresis in refractory neuro-irAEs. As immunotherapy becomes interwoven into early TNBC care, identifying predictive biomarkers and tailoring response-adapted regimens will be key to optimizing survival and quality of life.

## Figures and Tables

**Table 1:** Initial EMG demonstrating abnormality with the ulnar, median, and radial nerves.

Side	Muscle	Nerve	Root	Insertional Activity	Fibrillation Potentials	Positive Sharp Waves	Fasciculation	Amplitude	Duration	Primary Periodic Paralysis	Recruitment	Interference Pattern
Left	1st Dorsal Interosseous	Ulnar	C8–T1	Normal	3+	Normal	Normal	Normal	Normal	0	Reduce	25%
Left	Abductor Pollicis brevis	Median	C8–T1	Normal	3+	Normal	Normal	Normal	Normal	0	Reduce	25%
Left	Abductor Digiti minimi	Ulnar	C8–T1	Normal	3+	Normal	Normal	Normal	Normal	0	Reduce	25%
Left	Extensor digitorum communis	Radial	C7–8	Normal	Normal	Normal	Normal	Normal	Normal	0	Reduce	50%
Left	Flexor Digitoris Superior	Median	C7–8	Normal	Normal	Normal	Normal	Normal	Normal	0	Normal	Normal
Left	Biceps	Musculocutaneous	C5–6	Normal	Normal	Normal	Normal	Normal	Normal	0	Normal	Normal
Left	Triceps	Radial	C6–8	Normal	Normal	Normal	Normal	Normal	Normal	0	Normal	Normal
Left	Deltoid	Axillary	C5–6	Normal	Normal	Normal	Normal	Normal	Normal	0	Normal	Normal
Left	Flexor carpi ulnaris	Ulnar	C8–T1	Normal	2+	Normal	Normal	Normal	Normal	0	Normal	Normal
Left	Extensor indicis	Radial	C7–8	Normal	Normal	Normal	Normal	Normal	Normal	0	Normal	Normal

**Table 2:** Follow up EMG demonstrating resolution of the ulnar, median, and radial nerves after completing plasmapheresis.

Side	Muscle	Nerve	Root	Insertional Activity	Fibrillation Potentials	Positive Sharp Waves	Fasciculation	Amplitude	Duration	Primary Periodic Paralysis	Recruitment	Interference Pattern
Left	1st Dorsal interosseous	Ulnar	C8–T1	Normal	Normal	Normal	Normal	Normal	Normal	0	Normal	Normal
Left	Abductor pollicis brevis	Median	C8–T1	Normal	Normal	Normal	Normal	Normal	Normal	0	Normal	Normal
Left	Extensor digitorum communis	Radial	C7–8	Normal	Normal	Normal	Normal	Normal	Normal	0	Normal	Normal
Left	Biceps	Musculocutaneous	C5–6	Normal	Normal	Normal	Normal	Normal	Normal	0	Normal	Normal
Left	Triceps	Radial	C6–8	Normal	Normal	Normal	Normal	Normal	Normal	0	Normal	Normal

## Data Availability

The original contributions presented in this study are included in the article. Further inquiries can be directed to the corresponding author.

## Abbreviations

TNBC	Triple-negative breast cancer
N-IrAEs	Neurological immune-related adverse events
IrAEs	Immune-related adverse events
AI	Autoimmune
IVIG	Intravenous immunoglobulin
ICI	Immune checkpoint inhibitor
RIBP	Radiation-induced brachial plexopathy
TPE	Therapeutic plasma exchange
